# Are behavioral interventions a better choice for atopic dermatitis patients? A meta-analysis of 6 randomized controlled trials^☆^

**DOI:** 10.1016/j.abd.2023.09.004

**Published:** 2024-03-27

**Authors:** Wenying Zhong, Wei Li, Guangsheng Wu

**Affiliations:** a Department of Dermatology, The Affiliated Hospital of Hangzhou Normal University, Hangzhou, China; b Department of Pediatrics, The Affiliated Hospital of Hangzhou Normal University, Hangzhou, China

**Keywords:** Behavioral therapy, Cognitive behavioral therapy, Dermatitis, atopic, Meta-analysis

## Abstract

**Background:**

The treatment for atopic dermatitis (AD) has been the focus of clinical research, and behavioral intervention is considered an indispensable treatment method. To our knowledge, no relevant meta-analysis has evaluated the effects of behavioral interventions on atopic dermatitis.

**Objectives:**

To evaluate the effects of behavioral interventions on atopic dermatitis.

**Methods:**

The authors searched PubMed, EMBASE, and Cochrane CENTRAL to retrieve relevant RCTs (up to Feb 2022). The search strategy involved a combination of related keywords. The Cochrane Q and I^2^ statistics were used to assess heterogeneity.

**Results:**

Six RCTs involving seven reports with 246 patients were included. The results suggested that behavioral interventions could relieve eczema severity (correlation coefficient [*r* = −0.39]; p < 0.001) and scratching severity significantly (*r* = −0.19; p = 0.017), while not affect itching intensity (*r* = −0.02; p = 0.840). A sensitivity analysis confirmed the robustness of the results.

**Study limitations:**

An important limitation of this study was the insufficient number of RCTs and the limited sample size. In addition, the study lacked a control group receiving a type of intervention other than the experimental protocol. Another limitation was the short duration of follow-up.

**Conclusions:**

This study suggests that behavioral interventions could be effective in treating atopic dermatitis by reducing eczema and scratching severity. Additionally, habit-reversal behavioral therapy may be more effective for treating atopic dermatitis.

## Introduction

Atopic dermatitis is one of the most common chronic and relapsing inflammatory skin disorders worldwide, with a prevalence of 10%‒20% in children and 2%‒8% in adults.^1^, ^2^, ^3^ The clinical presentations of atopic dermatitis include disturbed epidermal differentiation and diminished skin barrier function.^4^ Atopic dermatitis is a potential contributor to cause debilitating symptoms, and it significantly reduces the patient’s quality of life.^5^, ^6^

Due to the heterogeneity in age, ethnicity, and lifestyle factors of patients, the etiology of atopic dermatitis has not been fully clarified.^7^, ^8^, ^9^ Currently, topical application of emollients and anti-inflammation agents is still the basic strategy for treating atopic dermatitis.^10^, ^11^ The topical therapy is not effective for the treatment among a considerable proportion of moderate-to-severe patients,^5^ which may cause a series of psychological consequences, such as sleep disturbance, anxiety, depression, and financial strain.^12^ Meanwhile, psychological factors have also been shown to aggravate skin symptoms.^13^ Therefore, psychological interventions are considered an indispensable element of multidisciplinary management strategy for atopic dermatitis.^14^, ^15^

Up to now, several original studies^16^, ^17^, ^18^ have investigated the therapeutic effects of various psychological intervention strategies on atopic dermatitis. Meanwhile, some meta-analyses^19^, ^20^ have also systematically evaluated the role of psychological and educational interventions in treating atopic dermatitis. However, the results of previously published studies of psychological interventions for atopic dermatitis are conflicting. It’s noted that the therapeutic effects of psychological interventions may change according to the variations of types of psychological interventions.^21^, ^22^ However, different psychological interventions were combined as an individual interventional strategy in the previous meta-analyses,^19^, ^20^ which may cause conflicting findings.

As an indispensable part of psychological interventions, behavioral therapy refers to the application of modern theories of learning and conditioning in the treatment of behavior disorders.^23^ Currently, many behavioral techniques have been used in clinical practice, such as cognitive behavioral therapy, habit-reversal therapy, and dialectical behavior therapy.^23^ Of these available behavioral techniques, habit-reversal therapy^24^ and cognitive behavioral therapy^25^, ^26^ have been used successfully in dermatology. Habit-reversal therapy was initially described by Azrin and Nunn as a competing response produced by tightening muscles with antagonists.^27^ Habit-reversal therapy consists of multiple components, but competing response training and awareness training represent its most effective techniques.^28^ While, cognitive behavioral therapy is a combination of cognitive and behavioral approaches, which can help the patient recognize his distorted thoughts and ineffective behaviors.^29^

Several studies^24^, ^26^ have explored the potential of habit-reversal therapy and cognitive behavioral therapy in managing atopic dermatitis. Bewley has reported that atopic dermatitis patients who received three weeks of habit-reversal therapy had significantly better atopic dermatitis scores than those of the topical treatments alone.^24^ Goyonlo et al. suggested that cognitive behavioral therapy helped dermatitis patients improve clinical severity scores.^26^ To our knowledge, there has been no relevant meta-analysis evaluating the effects of behavioral interventions on atopic dermatitis. Therefore, the authors conducted the present meta-analysis to assess the effects of separate behavioral interventions on health outcomes in atopic dermatitis patients. The authors aimed to provide a definitive evidence-based recommendation for behavioral interventions in atopic dermatitis patients.

## Methods

### Study design

This systematic review and meta-analysis was carried out following the structure recommended by the Cochrane Handbook,^30^ and was reported in strict accordance with the Preferred Reporting Items for Systematic Reviews and Meta-Analysis (PRISMA) checklist,^31^ thus strongly ensuring the transparency and reliability of conducting the current meta-analysis. All these fulfill the requirement of registering a formal protocol in a public platform, therefore the authors did not further register a protocol for this meta-analysis. This study did not require ethical approval and the patient’s informed consent.

### Selection criteria

Two independent reviewers strictly followed the criteria to select studies from the retrieved records. When there was disagreement about the literature retrieval, the authors resolved it by consulting a third reviewer. Three steps were designed for study selection: (a) Removal of duplicate studies based on EndNote software; (b) Initial eligibility assessment based on the titles and abstracts screening; and (c) Final eligibility assessment based on full-text evaluation.

#### Inclusion criteria

Eligibility of each study was evaluated using the PICOS acronym, including patients, intervention, comparison, outcome, and study design: (a) Patients were diagnosed with atopic dermatitis by a physician with recognized criteria (*P acronym*); (b) Patients in the experimental group were instructed to receive behavioral interventions in addition to usual medical care for atopic dermatitis (*I acronym*); (c) Patients in the control group received usual medical care alone (*C acronym*); (d) At least one of eczema severity, itching intensity, and scratching severity was reported effectively (*O acronym*); and (e) Patients were randomly assigned into different groups (*S acronym*).

#### Exclusion criteria

Studies were excluded from the following criteria: (a) Behavioral interventions were designed as one part of the psychological protocol rather than separate intervention; (b) Data about correlation coefficient (*r*) were not available; (c) Ineligible study design was used, such as quasi-randomized studies, observational studies, and animal studies; and (d) Duplicate studies with overlapped samples.

### Information sources

The authors systematically searched three common English databases, including PubMed, EMBASE, and the Cochrane Central Registry for Controlled Trials (CENTRAL), for retrieving relevant Randomized Controlled Trials (RCT) regarding the application of behavioral interventions in atopic dermatitis patients from their inception until February 2022.

### Search strategy

The search strategy was developed using subject terms and free words as follows: “atopic dermatitis”, “habit-reversal”, “behavior therapy”, “behavior modification”, “behavior change”, and “behavior treatment”. Two independent reviewers conducted the search and updated weekly to avoid missing potentially eligible studies. The authors also identified additional relevant studies through screening references of included studies and topic-related meta-analysis. The authors did not restrict the publication status in literature retrieval. Detailed search strategies of target databases were reported in Table S1.

### Data extraction

Two independent reviewers extracted the essential information from eligible studies using a pre-designed standard sheet. The following information was extracted: authors, year of publication, country, sample size, percentage of female patients, the mean age of patients, duration of atopic dermatitis, details of grouping, duration of intervention, the number of sessions, follow-up duration, detailed outcomes of interest, and information for quality assessment. The authors also contacted authors to obtain more information if necessary.

### Assessment of outcome

This meta-analysis evaluated three outcomes, including eczema severity, itching intensity, and scratching severity. Eczema severity was measured using the Scoring the Severity of Atopic Dermatitis (SCORAD), the Modified SCORAD, the AD Assessment Measure (ADAM), or the authors’ original scoring methods, and itching and scratching intensity was measured using a subjective Likert-type scale.

### Risk of bias assessment

Quality assessment was conducted by two independent reviewers using the Cochrane risk of bias assessment tool.^32^ In this assessment tool, seven items were involved, including random sequence generation (selection bias), allocation concealment (selection bias), blinding of participants and personnel (performance bias), blinding of outcome assessment (detection bias), incomplete outcome data (attrition bias), selective reporting bias (reporting bias), and other bias. Each item would be classified as “low”, “unclear”, or “high” risk based on the actual information reported in studies. Finally, the overall quality of each study was appraised as either “low”, “moderate”, or “high” level. Specifically, the overall methodological quality was appraised as “high” if all items were classified as “low” risk, “low” if at least one of the seven items was classified as “high” risk, or “moderate” if at least one of the seven items was classified as “unclear” risk but no one was classified as “high” risk.

### Statistical analysis

In this meta-analysis, the authors used Stata 14.0 software (State Corporation, Lake Way, Texas, USA) to calculate the effect size, which was expressed as a correlation coefficient (*r*).^33^ Statistical heterogeneity across studies was tested using Cochrane *Q*^34^, ^35^ and I^2^ statistic.^36^ Studies were considered homogeneous if p > 0.1 and I^2^ < 50%; Otherwise, they were regarded as heterogeneous. The authors selected the random-effects model for all statistical analyses regardless of the level of statistical heterogeneity because this model incorporated the variance resulting from differences between studies and within studies into estimates calculation.^35^ The authors calculated the corresponding *r* value according to the data reported in the included studies using an online calculator, namely the “Practical Meta-Analysis Effect Size Calculator”, which was designed by Wilson et al. to facilitate the computation of effect sizes for meta-analysis.^37^ The authors performed a sensitivity analysis to examine the robustness of results for eczema severity through the leave-one-out method. Finally, publication bias examination for meta-analysis of eczema severity was conducted using the Egger linear regression and Begg rank relationship methods although the cumulative number of eligible studies does not exceed 10.^38^

## Results

### Literature search

**Figure 1:**
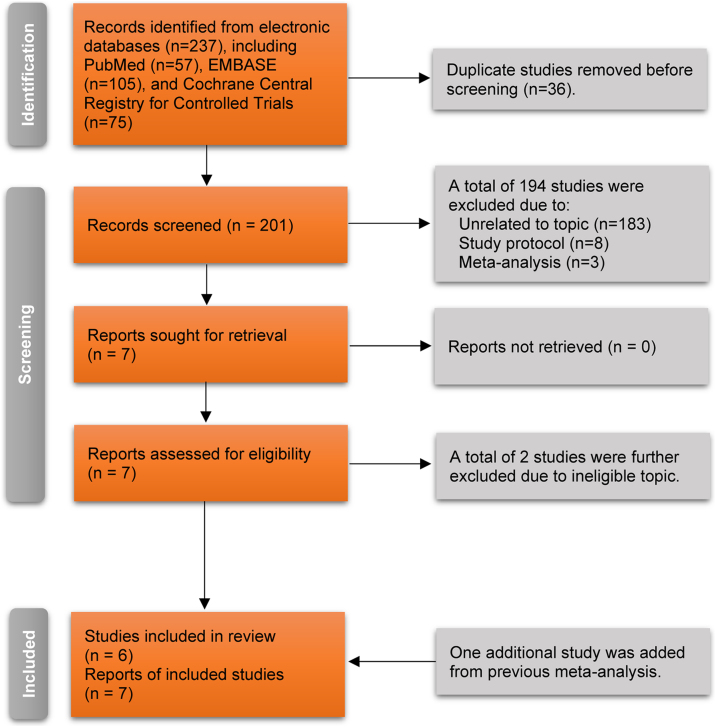
Flow chart of the process of selecting study.

The authors retrieved 237 records from electronic databases using pre-designed search strategies. After removing 36 duplicate records, 201 unique studies were retained for initial eligibility evaluation. The authors excluded a total of 194 ineligible studies after screening the titles and abstracts due to being unrelated to the topic (n = 183), registered study protocol (n = 8), and meta-analysis (n = 3). Then, the authors retrieved the full texts of the remaining seven studies for further eligibility evaluation. After excluding two ineligible studies due to ineligible topics, five studies^39^, ^40^, ^41^, ^42^, ^43^ were considered for meeting the selection criteria. Moreover, the authors identified another eligible study^44^ from a previous meta-analysis. Finally, six RCTs^39^, ^40^, ^41^, ^42^, ^43^, ^44^ involving seven reports were included in this meta-analysis because the study by Ehlers et al.^44^ compared the effectiveness of 4 group treatments for atopic dermatitis. The detailed process of selecting the study is indicated in Fig. 1.

### Study characteristics

Detailed basic characteristics of the included studies are presented in Table 1. A total of 246 patients were included in the current study.^39^, ^40^, ^41^, ^42^, ^43^, ^44^ Five studies^39^, ^40^, ^41^, ^42^, ^43^, ^44^ enrolled adult patients, and pediatric patients were included in only one study.^40^ Among six included studies, three studies^40^, ^41^, ^42^ designed habit reversal behavioral therapy, and another three^39^, ^43^, ^44^ designed cognitive behavioral therapy for treating atopic dermatitis. Three studies^40^, ^42^, ^44^ assigned physician to evaluate outcomes; two studies^41^, ^43^ measured outcomes with the Scoring Atopic Dermatitis (SCORAD); and one study^39^ used Atopic Dermatitis Assessment Measure (ADAM) to evaluate outcomes. The effect sizes of included studies regarding target outcomes of interest are presented in Table 2.

**Table 1:** The basic characteristics of the included studies.

Study	Country	Setting	Design	Sample size	Mean age, years	Percent female	Duration of disease	Duration of treatment	Number of sessions	Follow-up	Outcome assessment
Ehlers, et al., 1995	Germany	Clinic-based	UMC	19	22.3	68%	13.8	12 weeks	12	8 weeks	Physician’s evaluation
			DE	27	24.6	60%	15.3				
			BT	28	25.4	57%	15.7				
			DE + BT	27	25.4	62%	15.2				
Habib, et al., 1999	Australia	Population-based	UMC	8	33.0	87%	13.8	6 weeks	6	14 weeks	ADAM
			BT	9	36.0	78%	17.1				
Melin, et al., 1986	Sweden	Clinic-based	UMC	9	30.5	n.r.	N.R.	4 weeks	2	4 weeks	Physician’s evaluation
			BT	7							
Noren, et al., 1989	Sweden	Clinic-based	UMC	22	24.8	64%	N.R.	4 weeks	2	4 weeks	Physician’s evaluation
			BT	23							
Schut, et al., 2013	Germany	Population-based	UMC	14	22.3	71%	N.R.	5 weeks	5	8 weeks	SCORAD
			BT	14	23.6	71%	N.R.				
Noren, et al., 2018	Sweden	Population-based	UMC	21	8	72%	8.0	3 weeks	3	8 weeks	SCORAD
			BT	18	8	62%	6.0				

UMC, Usual Medical Care; DE, Dermatological Education; BT, Behavioral Therapy; ADAM, Atopic Dermatitis Assessment Measure; SCORAD, Scoring Atopic Dermatitis; N.R., Not Reported.

**Table 2:** Effect size of included studies regarding each outcome.

Study	Comparison	Eczema severity	Itching intensity	Scratching severity
Ehlers, et al., 1995a	BT vs. UMC	−0.348 (−0.575 to −0.071)	0.039 (−0.239 to 0.312)	−0.094 (−0.361 to 0.187)
Ehlers, et al., 1995b	DE + BT vs. DE	−0.328 (−0.546 to −0.069)	−0.073 (−0.326 to 0.189)	−0.109 (−0.358 to 0.154)
Habib, et al., 1999	BT vs. UMC	−0.499 (−0.790 to −0.024)	N.R.	N.R.
Melin, et al., 1986	BT vs. UMC	−0.426 (−0.761 to 0.088)	0 (−0.496 to 0.496)	−0.574 (−0.833 to −0.109)
Noren, et al., 1989	BT vs. UMC	−0.346 (−0.581 to −0.058)	N.R.	−0.248 (−0.505 to 0.049)
Schut, et al., 2013	BT vs. UMC	−0.272 (−0.586 to 0.113)	N.R.	N.R.
Noren, et al., 2018	BT vs. UMC	−0.518 (−0.721 to −0.233)	N.R.	−0.059 (−0.354 to 0.246)

UMC, Usual Medical Care; DE, Dermatological Education; BT, Behavioral Therapy; N.R., Not Reported.

### Risk of bias

**Figure 2:**
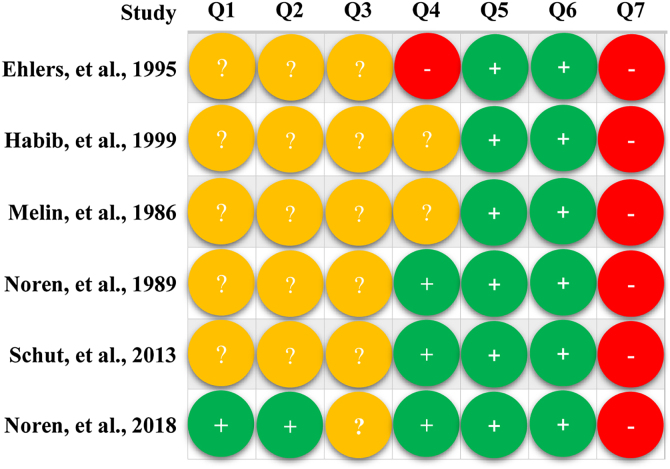
Risk of bias assessment of included studies. In this figure, Q1 to Q7 represents random sequence generation, allocation concealment, blinding of participants and personnel, blinding of outcome assessment, incomplete outcome data, selective reporting outcome, and other bias, respectively.

Only one study ^41^ specifically introduced the method of generating random sequences and performing allocation. All studies 39, 40, 41, 42, 43, 44 were rated as having an unclear risk for blinding participants and personnel, and one study ^44^ was rated as high risk for blinding of outcome assessment. All studies 39, 40, 41, 42, 43, 44 were rated as low risk for attrition bias and reporting bias but high risk for other biases due to extremely insufficient sample size. Detailed results of the quality assessment are presented in Fig. 2.

### Meta-analysis results

#### Eczema severity

**Figure 3:**
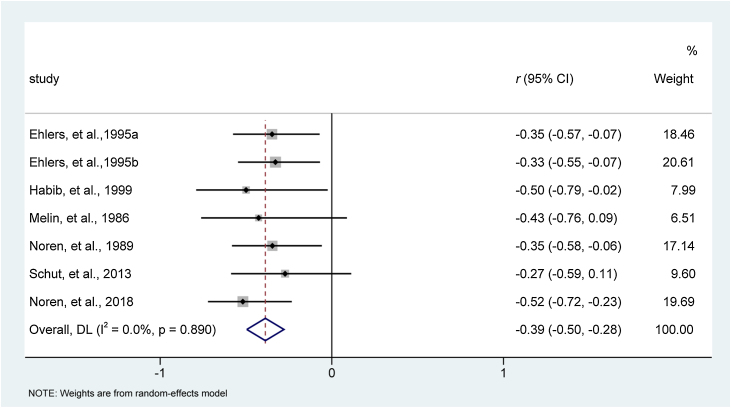
Meta-analysis of effect size of psychological interventions for relieving eczema severity. CI, Confidence Interval.

All included studies,^39^, ^40^, ^41^, ^42^, ^43^, ^44^ involving seven reports with 246 patients, evaluated the effect of behavioral interventions on eczema severity. Heterogeneity examination indicated that all studies were homogeneous for analysis of eczema severity (I^2^ = 0%, p = 0.890). Meta-analysis indicated that, compared to traditional medical care, behavioral interventions relieved eczema severity more effectively (effect size [95% CI]: −0.39 [−0.50, −0.28]; z = −7.003, p < 0.001). The forest plot is depicted in Fig. 3.

#### Itching intensity

**Figure 4:**
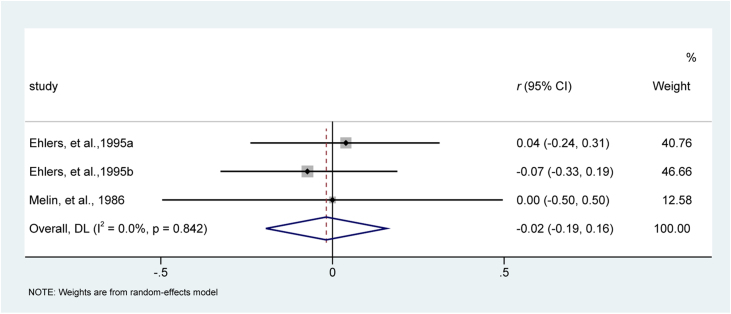
Meta-analysis of effect size of psychological interventions for reducing itching intensity. CI, Confidence Interval.

Among six included studies, two studies^40^, ^44^ involving three reports evaluated the effect of behavioral interventions on itching intensity. All studies were considered homogeneous for this analysis (I^2^ = 0%, p = 0.842). Meta-analysis suggested behavioral interventions were comparable with traditional medical care in terms of itching intensity, with a small effect size of −0.02 (95% CI −0.19 to 0.16; z = −0.202, p = 0.840). The forest plot is depicted in Fig. 4.

#### Scratching severity

**Figure 5:**
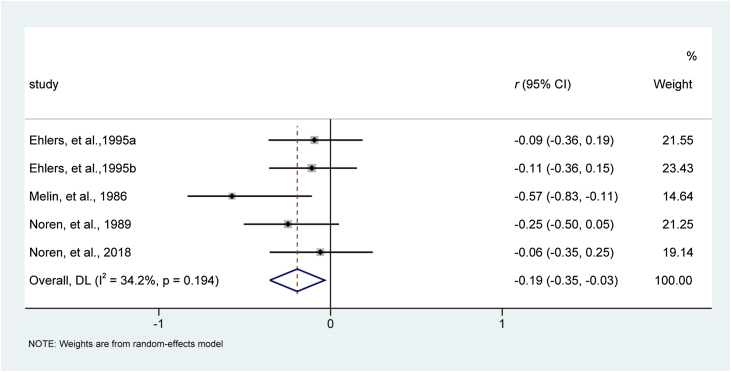
Meta-analysis of effect size of psychological interventions for reducing scratching severity. CI, Confidence Interval.

Four studies involving five reports^40^, ^41^, ^42^, ^44^ evaluated the effect of behavioral interventions on scratching severity. Heterogeneity examination did not detect substantial statistical heterogeneity across studies for this analysis of scratching severity (I^2^ = 34.2%, p = 0.194). Results of the meta-analysis suggested that behavioral interventions were associated with decreased scratching severity compared with usual medical care, with an effect size of -0.19 (95% CI −0.35 to −0.03; *z* = −2.377, p = 0.017). The forest plot is depicted in Fig. 5.

#### Subgroup analysis

As introduced above, two types of behavioral interventions were identified in this meta-analysis, including habit-reversal behavioral therapy and cognitive-behavioral therapy. We, therefore, conducted a subgroup analysis according to the types of interventions. Results from subgroup analysis suggested that both habit-reversal behavioral therapy and cognitive-behavioral therapy effectively relieved eczema severity, but only habit-reversal behavioral therapy significantly reduced scratching severity. Detailed results of subgroup analyses are presented in Figure S1.

#### Sensitivity analysis

To analyze eczema severity, the authors used the leave-one-out method to examine the robustness of the synthesized size of the effect. As presented in Figure S2, the synthesized size of the effect was not significantly changed after removing any one study at one time, indicating the robustness and credibility of the synthesized effect size in the current study.

#### Publication bias

The publication bias of studies included for analysis of eczema severity was evaluated using Egger’s test and Begg’s test. As shown in Figure S3, a symmetrical funnel plot was generated for Egger’s test (*z* = −0.15, p = 0.881) and Begg’s test (*t* = −0.19, p = 0.853), suggesting the absence of publication bias.

## Discussion

Although psychological interventions have been clinically recognized as effective, their benefits for treating atopic dermatitis are still controversial. This meta-analysis included 6 RCTs for data analysis. Results suggest that behavioral interventions are more effective than usual medical care in relieving eczema and scratching severity. Furthermore, habit-reversal behavioral therapy is a more efficacious intervention for treating atopic dermatitis because separate analysis further demonstrates its benefits in relieving eczema and scratching severity.

Currently, two published meta-analyses^19^, ^20^ evaluated the effects of psychological and educational interventions on atopic dermatitis. Chida et al.^19^ included eight studies in their meta-analysis, and they identified eight types of intervention: aromatherapy, autogenic training, brief dynamic psychotherapy, cognitive-behavioral therapy, dermatological education combined with cognitive-behavioral therapy, habit reversal behavioral therapy, stress management program, and structured educational programs. After calculating synthesized sizes of effect, they found that the role of psychological interventions in treating atopic dermatitis was premature, although it had a significant ameliorating effect on eczema severity, itching intensity, and scratching in atopic dermatitis patients. In 2017, Hashimoto et al. conducted another meta-analysis to investigate the effect of psychological and educational interventions on atopic dermatitis.^20^ Pooled results from 3 RCTs suggested no significant difference in eczema severity between the two groups. The effects of different psychological and educational interventions were different.^21^, ^22^ However, previously published two meta-analyses just incorporated different types of interventions into an individual regimen.

In this meta-analysis, the authors specifically investigated the pure effects of behavioral interventions on health outcomes in atopic dermatitis patients compared with previous meta-analyses. Meanwhile, the authors evaluated the effects of habit-reversal behavioral therapy and cognitive-behavioral therapy in treating atopic dermatitis, providing definitive evidence-based recommendations for clinical decision-making. More importantly, in this meta-analysis, the authors evaluated the role of behavioral interventions in treating atopic dermatitis by calculating the magnitude of therapeutic effect based on the correlation coefficient. Finally, the authors evaluated statistical heterogeneity across studies for the individual outcome. The authors calculated all synthesized sizes of effect with the random-effects model which simultaneously considered variations between studies and within studies. Therefore, the meta-analysis generated relatively conservative effect sizes.

However, the present meta-analysis remains to have some limitations. First and foremost, only six eligible studies with extremely insufficient sample sizes were included in this meta-analysis. Therefore, the current results should be cautiously interpreted due to inadequate statistical power. Second, patients enrolled in original studies were recruited from diverse backgrounds, including clinic-based and population-based backgrounds, which may introduce bias to the present findings. Third, five studies enrolled adult patients except for one study, in which pediatric patients were included. However, the authors performed a sensitivity analysis to examine the robustness of synthesized effects using the leave-one-out method. Sensitivity analysis indicated that behavioral interventions might be more effective for pediatric patients, but the pooled result did not change statistically. Fourth, the authors did not conduct a subgroup analysis to eliminate the effect of variations in the duration of disease, duration of treatment, number of sessions, duration of follow-up, and outcome assessment method due to the insufficient number of eligible studies. Fifth, as stated in the subsection of the study design, the authors did not register the used protocol in any public platform. However, the authors conducted this meta-analysis to ensure transparency and reliability in strict accordance with the Cochrane Handbook and PRISMA statements. Sixth, one study compared behavioral intervention combined with educational strategy to educational strategy alone; however, the authors also included it in the final analysis, which may introduce bias for the pooled results due to variations in control. Notably, sensitivity analysis based on the leave-one-out strategy suggested that the inclusion of this study did not significantly affect the reliability of the pooled results. Finally, although the examination for the analysis of eczema severity did not show publication bias risk, the authors must recognize that the cumulative number of eligible studies did not meet the minimum criteria of conducting a publication bias examination, it is therefore difficult to eliminate the risk of obtaining false negative results.

## Conclusion

In conclusion, based on the currently available evidence, the present meta-analysis indicates that behavioral interventions may be effective for relieving eczema severity and scratching severity. In addition, habit-reversal behavioral therapy may be more effective for the treatment of atopic dermatitis. However, the authors must recognize that the result for scratching severity is not accurate due to the small number of patients and the lower CI limits of almost null value. More importantly, the authors evaluated all outcomes only from a statistically significant perspective rather than from a clinical relevance. Therefore, the present findings should be further validated from a clinically relevant perspective in future studies.

## Financial support

This paper was supported by The Hangzhou Biomedical and health industry development support special project Research Fund (2021WJCY294). The funding bodies had no role in the design of the study and collection, analysis, and interpretation of data and in writing the manuscript.

## Authors’ contributions

Wenying Zhong: The study concept and design; Writing of the manuscript or critical review of important intellectual content; Data collection, analysis, and interpretation; Effective participation in the research guidance; Intellectual participation in the propaedeutic and/or therapeutic conduct of the studied cases; Critical review of the literature; Final approval of the final version of the manuscript.

Wei Li: Data collection, or analysis and interpretation of data; statistical analysis; Data collection, analysis, and interpretation; Intellectual participation in the propaedeutic and/or therapeutic conduct of the studied cases.

Guangsheng Wu: Data collection, or analysis and interpretation of data; Statistical analysis; data collection, analysis, and interpretation; Intellectual participation in the propaedeutic and/or therapeutic conduct of the studied cases.

## Conflicts of interest

None declared.
