# Supplementary material for: Are behavioral interventions a better choice for atopic dermatitis patients? A meta-analysis of 6 randomized controlled trials
Source: An Bras Dermatol. 2024 Mar 27;99(4):503–12. doi: 10.1016/j.abd.2023.09.004 (PMC11220917; doi:10.1016/j.abd.2023.09.004)

ABD-D-23-00336_Supplementary Material

**Supplemental Table S1** Search strategy.

| **Search strategy for PubMed** | | | | |
| --- | --- | --- | --- | --- |
| **Search number** | **Query** | **Sort By** | **Filters** | **Results** |
| 10 | (("Dermatitis, Atopic"[Mesh]) OR ((((((((Atopic Dermatitides[Title/Abstract]) OR (Atopic Dermatitis[Title/Abstract])) OR (Atopic Neurodermatitides[Title/Abstract])) OR (Atopic Neurodermatitis[Title/Abstract])) OR (Disseminated Neurodermatitides[Title/Abstract])) OR (Disseminated Neurodermatitis[Title/Abstract])) OR (Atopic Eczema[Title/Abstract])) OR (Infantile Eczema[Title/Abstract]))) AND (("Behavior Therapy"[Mesh]) OR ((((((((((Behavior Therapies[Title/Abstract]) OR (Behavior Treatment[Title/Abstract])) OR (Conditioning Therapy[Title/Abstract])) OR (Conditioning Therapies[Title/Abstract])) OR (Behavior Change Techniques[Title/Abstract])) OR (Behavior Change Technique[Title/Abstract])) OR (Behavior Modification[Title/Abstract])) OR (Behavior Modifications[Title/Abstract])) OR (habit-reversal[Title/Abstract])) OR (habit reversal[Title/Abstract]))) | Most Recent | | 57 |
| 9 | ("Behavior Therapy"[Mesh]) OR ((((((((((Behavior Therapies[Title/Abstract]) OR (Behavior Treatment[Title/Abstract])) OR (Conditioning Therapy[Title/Abstract])) OR (Conditioning Therapies[Title/Abstract])) OR (Behavior Change Techniques[Title/Abstract])) OR (Behavior Change Technique[Title/Abstract])) OR (Behavior Modification[Title/Abstract])) OR (Behavior Modifications[Title/Abstract])) OR (habit-reversal[Title/Abstract])) OR (habit reversal[Title/Abstract])) | Most Recent | | 87,033 |
| 8 | (((((((((Behavior Therapies[Title/Abstract]) OR (Behavior Treatment[Title/Abstract])) OR (Conditioning Therapy[Title/Abstract])) OR (Conditioning Therapies[Title/Abstract])) OR (Behavior Change Techniques[Title/Abstract])) OR (Behavior Change Technique[Title/Abstract])) OR (Behavior Modification[Title/Abstract])) OR (Behavior Modifications[Title/Abstract])) OR (habit-reversal[Title/Abstract])) OR (habit reversal[Title/Abstract]) | Most Recent | | 4,458 |
| 7 | "Behavior Therapy"[Mesh] | Most Recent | | 84,194 |
| 3 | ("Dermatitis, Atopic"[Mesh]) OR ((((((((Atopic Dermatitides[Title/Abstract]) OR (Atopic Dermatitis[Title/Abstract])) OR (Atopic Neurodermatitides[Title/Abstract])) OR (Atopic Neurodermatitis[Title/Abstract])) OR (Disseminated Neurodermatitides[Title/Abstract])) OR (Disseminated Neurodermatitis[Title/Abstract])) OR (Atopic Eczema[Title/Abstract])) OR (Infantile Eczema[Title/Abstract])) | Most Recent | | 32,130 |
| 2 | (((((((Atopic Dermatitides[Title/Abstract]) OR (Atopic Dermatitis[Title/Abstract])) OR (Atopic Neurodermatitides[Title/Abstract])) OR (Atopic Neurodermatitis[Title/Abstract])) OR (Disseminated Neurodermatitides[Title/Abstract])) OR (Disseminated Neurodermatitis[Title/Abstract])) OR (Atopic Eczema[Title/Abstract])) OR (Infantile Eczema[Title/Abstract]) | Most Recent | | 26,933 |
| 1 | "Dermatitis, Atopic"[Mesh] | Most Recent | | 22,495 |

| **Search strategy for Embase** | | |
| --- | --- | --- |
| **Nº** | **Query** | **Results** |
| #8. | #7 AND [embase]/lim | 105 |
| #7. | #3 AND #6 | 108 |
| #6. | #4 OR #5 | 75,276 |
| #5. | 'behavior therapy'/exp | 67,508 |
| #4. | 'behavior therapies':ti,ab,kw OR 'behavior treatment':ti,ab,kw OR 'conditioning therapy':ti,ab,kw OR 'conditioning therapies':ti,ab,kw OR 'behavior change techniques':ti,ab,kw OR 'behavior change technique':ti,ab,kw OR 'behavior modification':ti,ab,kw OR 'behavior modifications':ti,ab,kw OR 'habit reversal':ti,ab,kw OR 'behavior therapy':ti,ab,kw | 14,207 |
| #3. | #1 OR #2 | 56,522 |
| #2. | 'atopic dermatitis'/exp | 50,945 |
| #1. | 'atopic dermatitides':ti,ab,kw OR 'atopic dermatitis':ti,ab,kw OR 'atopic neurodermatitides':ti,ab,kw OR 'atopic neurodermatitis':ti,ab,kw OR 'disseminated neurodermatitides':ti,ab,kw OR 'disseminated neurodermatitis':ti,ab,kw OR 'atopic eczema':ti,ab,kw OR 'infantile eczema':ti,ab,kw | 43,073 |

| **Search strategy for Cochrane Central Registry for Controlled Trials** | | |
| --- | --- | --- |
| **ID** | **Search** | **Hits** |
| #1 | (Atopic Dermatitides):ti,ab,kw OR (Atopic Dermatitis):ti,ab,kw OR (Atopic Neurodermatitides):ti,ab,kw OR (Atopic Neurodermatitis):ti,ab,kw OR (Disseminated Neurodermatitides):ti,ab,kw | 5176 |
| #2 | (Disseminated Neurodermatitis):ti,ab,kw OR (Atopic Eczema):ti,ab,kw OR (Infantile Eczema):ti,ab,kw | 2403 |
| #3 | #1 or #2 | 5487 |
| #4 | MeSH descriptor: [Dermatitis, Atopic] explode all trees | 1959 |
| #5 | #3 or #4 | 5487 |
| #6 | (habit-reversal):ti,ab,kw OR (habit reversal):ti,ab,kw OR (Behavior Therapy):ti,ab,kw OR (Behavior Therapies):ti,ab,kw OR (Behavior Treatment):ti,ab,kw | 49738 |
| #7 | (Conditioning Therapy):ti,ab,kw OR (Conditioning Therapies):ti,ab,kw OR (Behavior Change Techniques):ti,ab,kw OR (Behavior Change Technique):ti,ab,kw OR (Behavior Modification):ti,ab,kw | 7691 |
| #8 | (Behavior Modifications):ti,ab,kw | 675 |
| #9 | #6 or #7 or #8 | 54230 |
| #10 | MeSH descriptor: [Behavior Therapy] explode all trees | 18389 |
| #11 | #9 or #10 | 64283 |
| #12 | #5 and #11 | 76 |

**Figure S1** Subgroup analysis for eczema severity, itching intensity, and scratching severity according to the types of psychological interventions. CI, Confidence Interval.


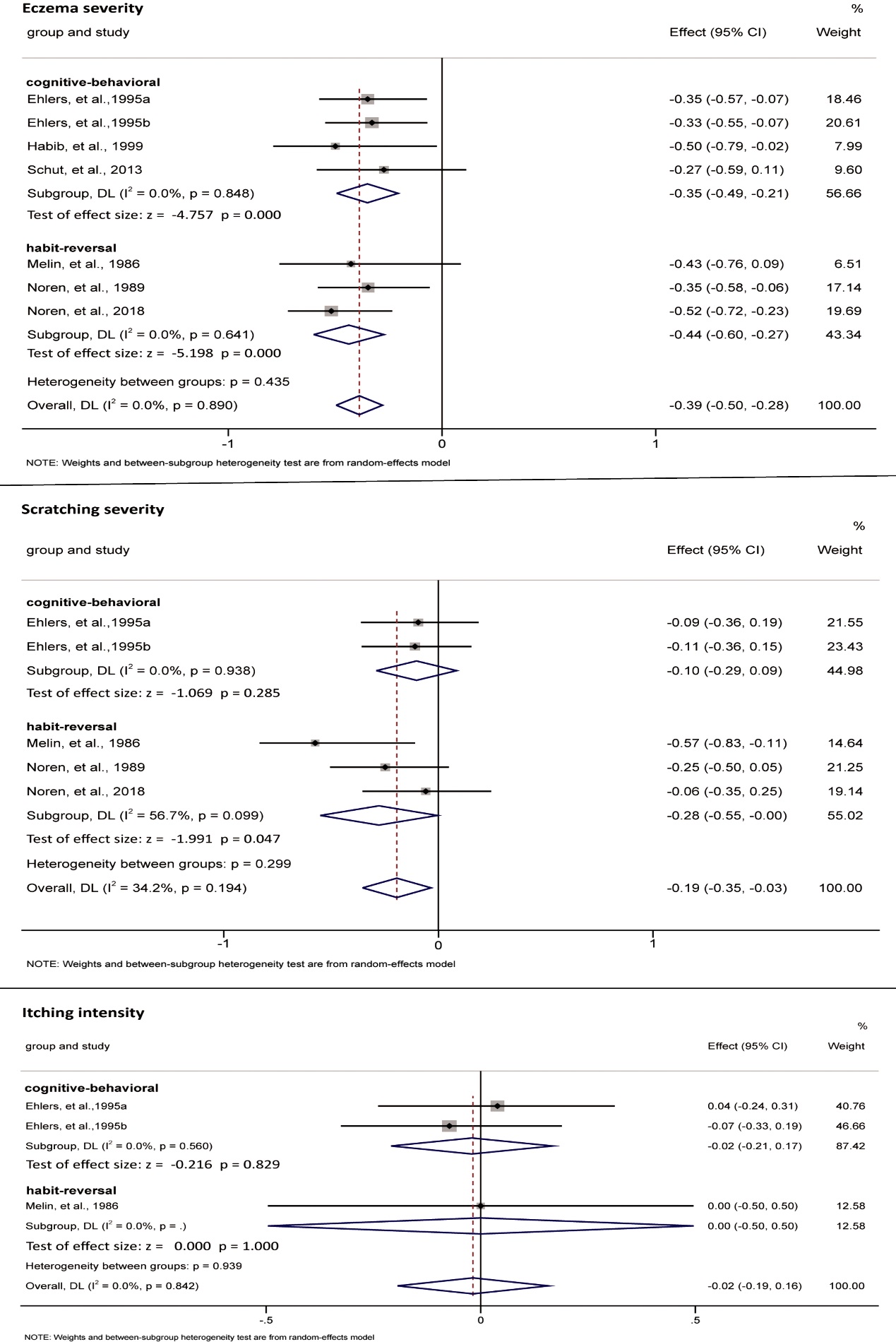


**Figure S2** Sensitivity analysis for eczema severity based on leave-one-out method. CI, Confidence Interval.


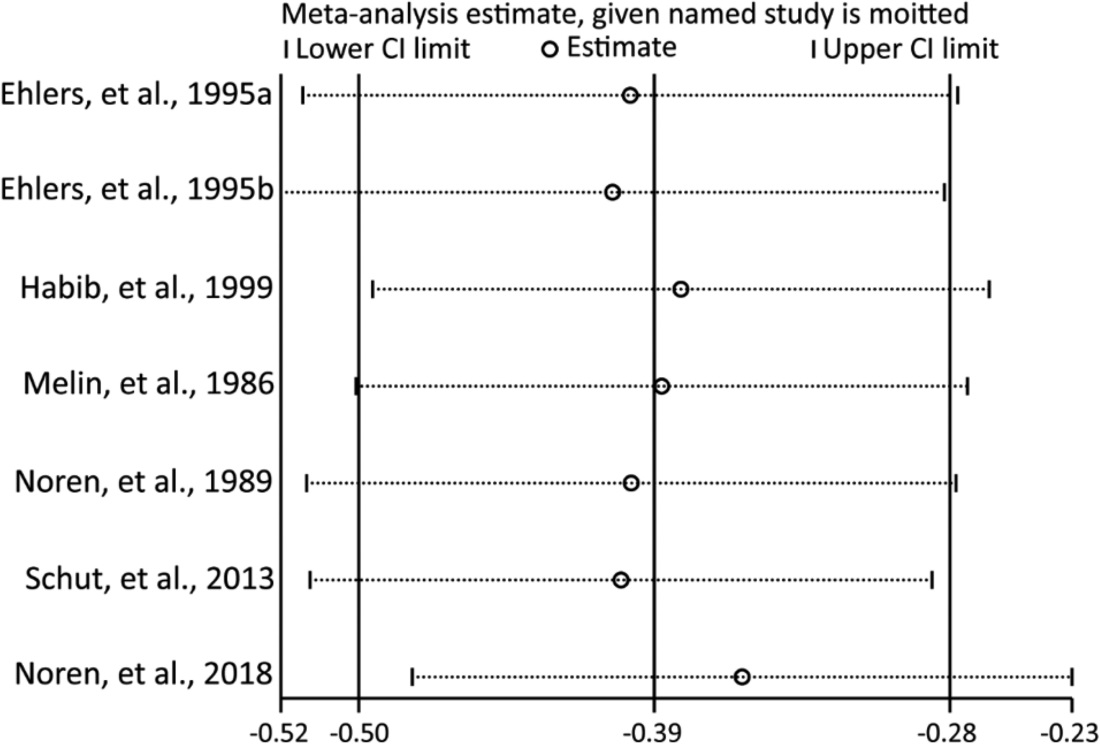


**Figure S3** Publication bias examination for eczema severity based on Egger’s test and Begg’s test.


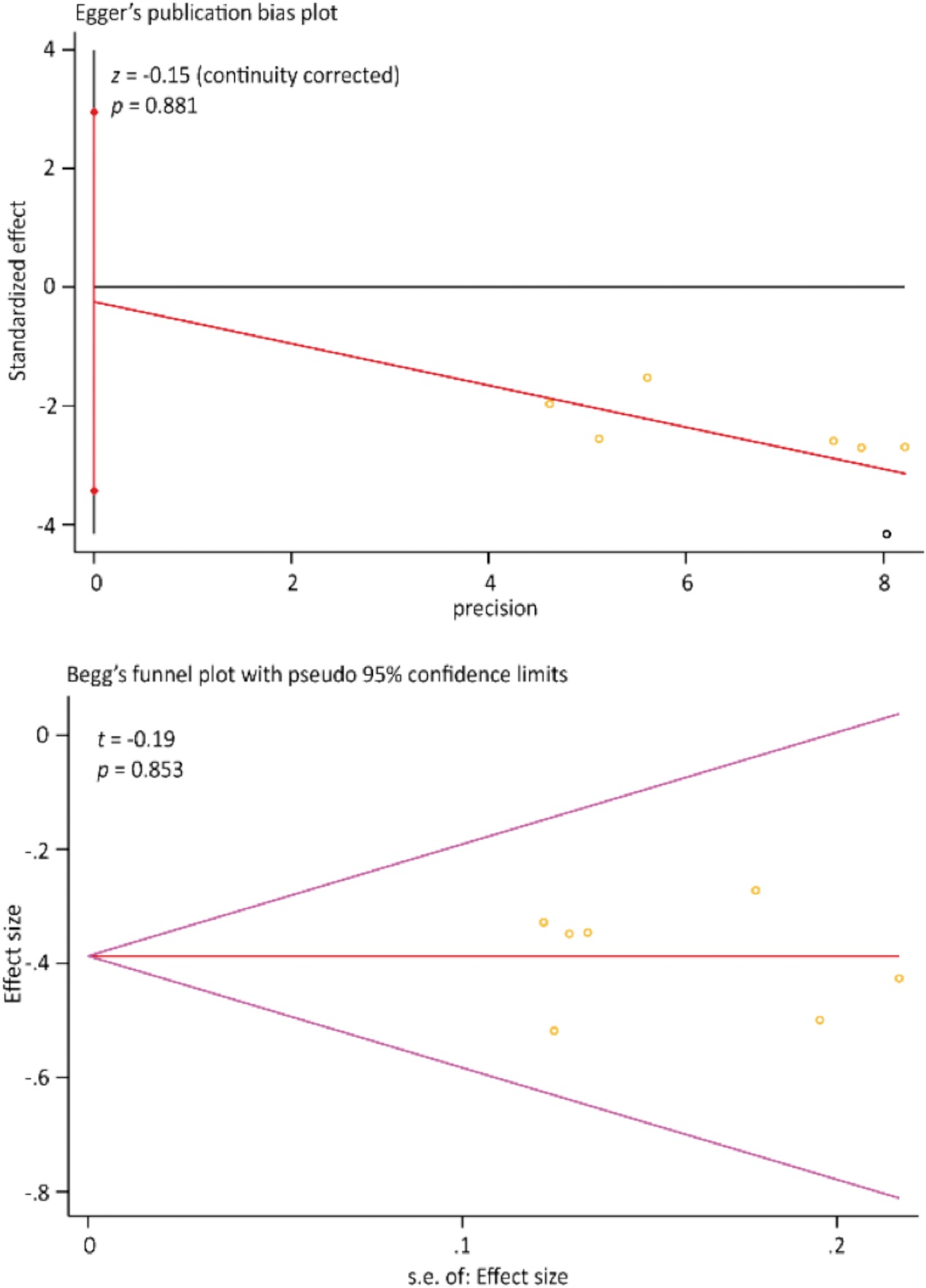

Supplement: Supplementary file 1 [file mmc1.docx]
